# Multilevel selection of *bcrABDR*-mediated bacitracin resistance in *Enterococcus faecalis* from chicken farms

**DOI:** 10.1038/srep34895

**Published:** 2016-10-12

**Authors:** Mu-Ya Chen, Felipe Lira, Hua-Qing Liang, Rui-Ting Wu, Jia-Hong Duan, Xiao-Ping Liao, José L. Martínez, Ya-Hong Liu, Jian Sun

**Affiliations:** 1 National Risk Assessment Laboratory for Antimicrobial Resistance of Animal Original Bacteria, South China Agricultural University, Guangzhou, China; 2 Guangdong Provincial Key Laboratory of Veterinary Pharmaceutics Development and Safety Evaluation, South China Agricultural University, Guangzhou, China; 3 Centro Nacional de Biotecnología, CSIC, Darwin 3, Madrid-28049, Spain

## Abstract

In this study we isolated 109 *Enterococcus faecalis* from chicken faecal samples in 6 provinces of China to investigate the prevalence and transmission mechanism of the bacitracin resistance locus *bcrABDR* in *E. faecalis*. Thirty-seven *bcrABDR*-positive *E. faecalis* were detected with 26 different PFGE clusters. The MLST of 14 positive strains belonged to ST16 and we also detected three new sequence types. S1-PFGE analysis indicated that the locus was located on plasmids presenting different sizes, with the most prevalent size being ~50 kb (13/37). Sequence analysis revealed that 17 out of the 37 strains harbored a 5400-bp central region, in which locus *bcrABDR* was bracketed by two IS*Enfa1* of the same orientation. Two types of *bcrABDR* alleles, differing in around 10% of their sequence were found. In silico analysis showed that *bcrABDR* is present in a variety of bacteria including the chicken commensal *Enterococcus cecorum*. Our results indicate that the use of bacitracin at farms might trigger the emergence and spread of the bacitracin resistance determinant *bcrABDR* among human bacterial pathogens. The finding of *bcrABDR* in the chicken commensal *E. cecorum* indicates that farm animals microbiota can be an important reservoir of resistance genes with relevance for human health.

Bacitracin is a peptide antimicrobial that inhibits the cell wall biosynthesis by binding to the lipid undecaprenyl pyrophosphate (UPP) and impeding its dephosphorylation. UPP is the precursor of the undecaprenol monophosphate that serves as a membrane-associated carrier for sugar-peptide units in the peptidoglycan synthesis. After completion of the cycle, UPP is dephosphorylated to form undecaprenol monophosphate (UP) to start another synthetic cycle^1^. By binding UPP, bacitracin reduces the amount of available UP, stopping the synthesis of peptidoglycan, which finally leads to bacterial death.

It has been reported that bacitracin can be an effective antibiotic to treat patients who are infected with vancomycin-resistant enterococci (VRE)^2^,^3^. Nevertheless, this antibiotic is also used as animal growth promoter in China, and this wide use of the bacitracin for non-therapeutic procedures might compromise its utility for human health. Indeed, some recent reports have shown that high-level bacitracin resistance (MIC ≥ 256 μg/ml) in enterococci is becoming frequent and widespread^4^. Several bacitracin resistance mechanisms have been described in Gram-negative and Gram-positive bacteria^5^,^6^. In *E. faecalis*, a primary resistance mechanism has been reported to be the presence of the *bcrABDR* cluster, composed by the *bcrABD operon* and its regulatory gene *bcrR*^7^. The *bcrABD* operon encodes a putative heterodimeric ATP-binding cassette (ABC) transporter BcrAB that has been proposed to mediate the active efflux of bacitracin^8^. In addition, BcrD is a putative undecaprenol kinase that would convert UPP to UP. Overproduction of the undecaprenol kinase increases the levels of UP, which can overcome the low levels of UPP when the pathway is blocked by bacitracin^9^. Finally, BcrR has been identified as a bacitracin able sensor to regulate the expression of the operon *bcrABD*^10^.

In *E. faecalis*, the *bcrABDR* cluster has been sporadically detected^11^, but little is known about its prevalence and genetic environment. Our aim is to investigate the prevalence of the *bcrABDR* cluster in *E. faecalis* isolates obtained from animal samples from six different provinces in China in the aim of determining whether or not the wide use of bacitracin in farms may render a selection of strains carrying this resistance determinant, which might compromise the use of bacitracin in human therapy. In addition, we have explored the different hierarchical levels (from genes to clones) at which selection of resistance may operate in the aim of defining the elements involved in the allodemic^12^ spread of this resistance determinant, and its consequences for human health.

## Results

### The bacitracin resistance locus *bcrABDR* is widespread in *Enterococcus faecalis* isolates from chickens

**Figure 1:**
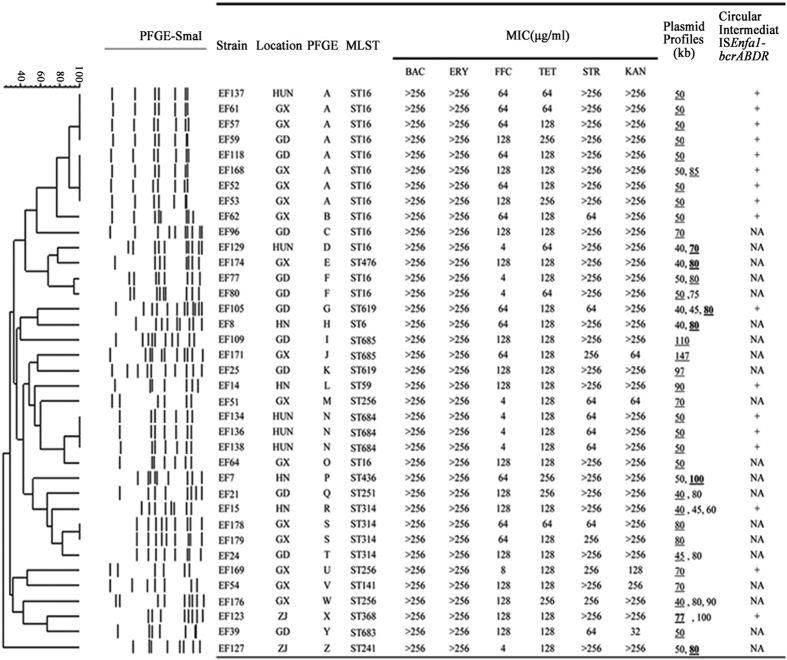
The information of *bcrABDR*-positive strain. The Figure shows epidemiological, phylogenetical and antibiotic resistance information of the strains analyzed in the current article. Strain EF21, EF24 and EF176 carry the locus *bcrABDR* highly similarity with the respect gene reported in plasmid pTW9 (NC_014726); ST683, ST684 and ST 685 were new ST types summation in this study; The size for locus *bcrABDR* positive plasmid is indicated by underline; The size for conjugative plasmid is indicated by black. NA, not available; UN, Hunan; GX, Guangxi; GD, Guangdong; ZJ Zhejiang; HN, Hainan; BAC, bacitracin; STR, streptomycin; ERY, erythromycin; TET, Tetracycline; FFC, florfenicol; KAN, kanamycin.

109 *E. faecalis* from 11 farms in 6 provinces of China (Table S1) were studied to determine the incidence and mechanisms of bacitracin resistance associated to farming activities. Among them, 37 (33.9%) strains displayed high-level resistance (MIC ≥ 256 μg/ml) to bacitracin. PCR analysis demonstrated that all the 37 isolates carried *bcrABDR*. In addition to presenting bacitracin resistance, all the *bcrABDR*-positive isolates were resistant to erythromycin, tetracycline, and streptomycin. Additionally, most of them were resistant to florfenicol and kanamycin (Fig. 1).These data indicate that bacitracin resistance, associated to the presence of *bcrABDR* is highly prevalent in *E. faecalis* strains isolated from chicken at farms in China.

### The acquisition of *bcrABDR* is polyclonal in *E. faecalis*

The 37 *bcrABDR*-positive *E. faecalis* isolates displayed different PFGE patterns and belonged to 26 clusters designated A to Z (Fig. 1). Cluster A, F, N, S contained 8, 2, 3, and 2 isolates respectively, and the other clusters contained a single strain each. MLST results showed a diversity of STs of the 37 isolates, with 14 strains belonging to ST 16. We also detected three new ST types, including ST683, ST684 and ST685 (Fig. 1).

### Different transferrable plasmids are involved in the spread of *bcrABDR* in *E. faecalis*

Seven plasmids carrying *bcrABDR* were successfully transferred to a susceptible recipient strain through conjugation experiments. When compared with the recipient strain, the bacitracin MICs of the seven transconjugants increased at least 8-fold. In addition to bacitracin, co-transfer of resistance, erythromycin, streptomycin and kanamycin, were also observed in these seven transconjugants. Further, five of them conferred as well resistance to tetracycline and two transferred florfenicol resistance (Table S3). Altogether, these results highlight that *bcrABDR*-mediated bacitracin resistance is present in multi-resistance plasmids, supporting that co-selection by one or another of the antibiotics to which the plasmids confer resistance might be in the basis of their high prevalence.

To further characterize the plasmids carrying the *bcrABDR* operon, S1-PFGE followed by Southern blot using a *bcrB*-specific probe was performed for all the isolates containing *bcrABDR*. As shown in (Fig. S1), only a single plasmid hybridized with the *bcrB*-specific probe for each of the analyzed strains. The plasmids harboring the *bcrABDR* operon presented sizes ranging from 40 kb to 147 kb, with ~50kb being the most the most frequent size (13/37) (Figs 1 and S1).

To gain further insight into the mechanisms mediating *bcrABDR* spread, one of the plasmids (pEF123) was fully sequenced as described in methods. As shown in (Fig. S2), *bcrABDR* is surrounded in pEF123 by two IS*Enfa1* insertion sequences, a structure also observed for the *bcrABDR*-containing plasmids pXD5 and pTW9. Notably, several other plasmids that do not contain the *bcrABDR* operon also presented IS*Enfa1*, indicating that this IS might be critical elements for the acquisition of genetic material and the evolution of *E. faecalis* plasmids.

### The genetic environment surrounding *bcrABDR* is not always the same in conjugative plasmids

**Figure 2:**
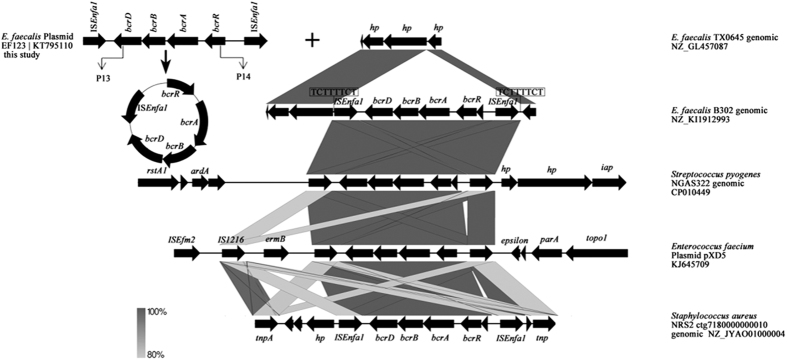
Genetic environment of the locus *bcrABDR* on the plasmid JEF123. The genetic environment of *bcrABDR* from the *E. faecalis* EF123 analyzed in the study is shown in comparison with *E. faecalis* TX0645 genomic, *E. faecalis* B302 genomic, *Streptococcus pyogenes* NGAS322 genomic, *Enterococcus faecium* plasmid pXD5, *Staphylococcus aureus* NRS2 ctg7180000000010 genomic sequences. The positions and orientations of the genes are indicated by arrows, with the direction of transcription shown by the arrowhead. Regions of homology are shaded in gray. The whole IS*Enfa1*-*bcrABDR*-IS*Enfa1* segment is 5400 bp in length. The 8 bp direct target site duplication sequence (5-TCTTTTCT-3) is boxed. The black arrows indicated the positions and orientations of the inverse PCR primers P13/P14.

The regions surrounding the *bcrABDR* operon were determined by primer walking. Three different genetic environments (designated as type I–III) were detected in the 7 transconjugants. In the type I genetic organization, the locus *bcrABDR* was flanked by two copies of the insertion sequence IS*Enfa1* located in the same orientation (JEF105, JEF123) and the locus *bcrABDR* was oriented in the opposite direction of IS*Enfa1*. To determine the stability of this structure, inverse PCR was performed using the primersP13/P14. Sequence analysis of the PCR production revealed that it contained one intact IS*Enfa1*, supporting that *bcrABDR* might form a circular intermediate through the recombination of the two copies of IS*Enfa1*. Figure 2 The inverse PCR was also performed on all the *bcrABDR*-positive strains, and 17 of them gave a positive result. Together with previously published works^13^, our results further support that the formation of circular intermediates may mediate the transfer of *bcrABDR*. In the type II organization, IS*Enfa1* was only located upstream the locus *bcrABDR* (JEF7, JEF8, JEF127 and JEF129) without any IS*Enfa1* located downstream. In the type III organization we could not find the insertion sequence IS*Enfa1* neither upstream nor downstream *bcrABDR* (JEF174). Although it is tempting to propose that these results suggest a route for the evolution and spread of *bcrABDR* from bacteria not harboring IS*Enfa1* towards strain carrying one IS*Enfa1* copy, ending in those that carry two IS*Enfa1* copies, in which the circular intermediate can be found, the opposite situation might also happens if loss of IS*Enfa1* occur through abortive recombination events.

### The origin of plasmid-encoded *bcrABDR* is polyphyletic in *E. faecalis*

**Figure 3:**
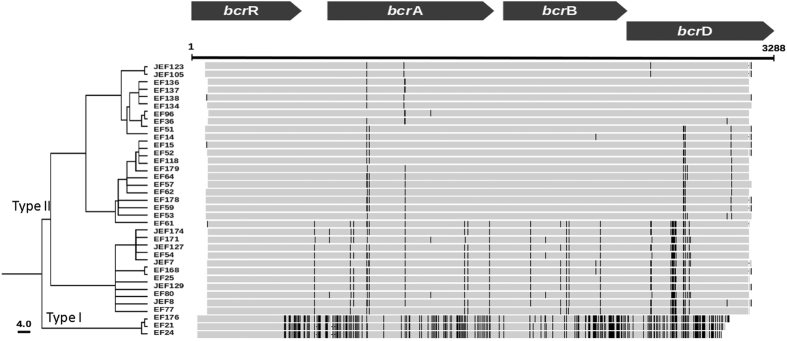
Phylogenetic relationship of *bcrABDR* clusters analyzed in the present work. The figure shows the phylogenetic relationships among the different *bcrABDR* alleles studied in the present work. As shown, two major branches (Type I and Type II) can be distinguished, with the Type II allele presenting also three different subtypes. This population structure indicates that the acquisition of *bcrABDR* by *E. faecalis* has a polyphyletic origin. Each black line in the Figure represents a SNP in comparison with the consensous *bcrABDR* sequence.

From the 37 *bcrABDR*-positive isolates, the four genes in the locus could be amplified just in three of the isolates with the primers previously described. For the other 34 isolates the genes *bcrA* and *bcrD* could not be detected using these primers, so new primers for these genes were designed according to the sequence of the plasmid pEF123 determined in this study by using these novel primers (Table S2), a new allelic variant of the *bcrABDR* operon was found. The amino acid sequences of BcrA, BcrB, BcrD and BcrR in the two alleles presented similarities of 90.8%, 88.9%, 88.3% and 94.1% respectively. Further, the Type II allele presents at least three different subtypes that were similar among them, but displayed some few consistent different SNPs (Fig. 3). Independent Blast searches using the two different allelic forms of the *bcrABDR* operon were performed. By means of these approaches, we found that both types of sequences are present at the NCBI database. The firstly described *bcrABDR* allele (hereafter dubbed as Type I), was found and identity of 99% in pTW9 and pJM01, whereas the *bcrABDR* sequence present in pEF123 (Type II), with a 99% identity was found in the plasmids pXD5 and R17. It has been discussed that the low variability of antibiotic resistance genes found in the mobile gene elements of bacterial pathogens might be the consequence of a founder effect^14^. The first resistance gene to spread will prevail and impede the entrance of other resistance elements rendering the same phenotype. Here we detect that two *bcrABDR* alleles (or maybe more if we consider the Type II subtypes as different alleles) are present in bacitracin resistant *E. faecalis* indicating that in this case at least two and likely four, different events might account for the acquisition of resistance. This situation may happen when there exists a close ecological relationship between the donor and the recipient of the resistance element, which allows these transfer events to occur frequently and being efficiently selected, likely as the consequence of bacitracin use in chicken farms.

### *Enterococcus cecorum* can be a reservoir of bacitracin resistance in chicken

To ascertain the distribution of *bcrABDR*, the sequence of the cluster and the surrounding IS*Enfa1* was searched at NCBI DNA database. In addition to the aforementioned plasmids, Type II *bcrABDR* was found to be present in the genome of *Streptocccus pyogenes* NGAS322 as well as in the genomes of different strains of *E. cecorum* (a chicken commensal with some virulent members) isolates^15^,^16^,^17^, in occasions surrounded by IS*Enfa1*, in other occasions without IS present around. In addition, the genomes of *E. cecorum* also contain upstream and downstream sequences nearly identical to those found in pEF123 and other *bcrABDR*-containing plasmids. All of these will be powerful to improve *Enterococcus cecorum* can be a reservoir of bacitracin resistance in chicken. Although the whole-genome sequence methods used in this study did not allow distinguishing between chromosomal and plasmidic DNA. Since a route of transmission from *E. cecorum* towards *E. faecalis* or *vice versa* cannot be tracked, we cannot ascertain which is the origin of *bcrABDR*; however our results indicate that the commensal chicken microbiota can be a reservoir of transferrable bacitracin resistance that may end in relevant human pathogens as *E. faecalis.*

## Discussion

In this study, we investigated the prevalence of the *locus bcrABDR* in *E. faecalis* isolated from 6 provinces in China. Our study showed that resistance to bacitracin, associated to the presence of plasmids containing the *bcrABDR* cluster was highly prevalent and associated with resistance to other antimicrobials, especially erythromycin, streptomycin, kanamycin, florfenicol and tetracycline. The polyclonal phenomenon result in *E. faecalis* indicated that different clones are involved in the spread of bacitracin resistant *E. faecalis* isolates containing the *bcrABDR* cluster. Persistence and co-selection of the *bcrABDR* cluster could be promoted under antibiotic selective pressure, when these classes of antimicrobials were used either as feed additives or to cure or prevent bacterial infections in animals^18^. When looking at the different elements, from genes to clones that may be involved in the spread of this resistance, we found that at least two different alleles of the *bcrABDR* cluster are present in different plasmids, suggesting the existence of multiple events of acquisition (and fixation) of this resistance determinant by *E. faecalis* from its (still unknown) original host. After these acquisition events, the spread of *bcrABDR* has been guided by its incorporation in different plasmids, which are also spread among different clonal complexes.

The inspection of the *bcrABDR* flanking regions showed three different organizations, in most cases, two intact IS*Enfa1* flanked the *bcrABDR* cluster and inverse PCR shows that they can form *bcrABDR*-containing circular intermediates through homologous recombination, which suggests that IS-mediated recombination may account for the transfer of the *bcrABDR* cluster between different genetic platforms. Interestingly the structure IS*Enfa1*-*bcrABDR*-IS*Enfa1* is also observed in other bacterial species, which can further suggest that the form of the circular intermediate is the main manner for the transmission of the locus *bcrABDR* (Fig. 2). For the other two types of genetic environments, in which *bcrABDR* is not flanked by IS*Enfa1* the mechanisms involved in its acquisition and spread are not known at the moment.

All these results indicated that multiple levels of selection regulate the spread of *bcrABDR* among *E. faecalis* populations. The fact that this operon presented in other species^13^,^19^ shows that inter-species transmission is involved in the spread of this element. In this regard, it is particularly relevant that the *bcrABDR* operon is found in *E. cecorum*, which is the dominant enteric commensal of adult chicken and contributes to the gut microbiota of several avian species. This indicated that the commensal microbiota of farming animals can be a reservoir of antibiotic resistance that can be transferred to human pathogens. Indeed, MLST typing revealed that 14 of 37 *bcrABDR*-positive strains belonged to the high-risk clone ST16, which has been reported to be associated with the prevalence of the multi-resistance gene *cfr* in *E. faecalis* and VRE^20^,^21^ and is commonly found in animals, humans, and the environment^22^. The presence of ST16 in both poultry and faecal samples of healthy humans had been observed in Portugal, Denmark, New Zealand, Japan, Thailand, and the USA^23^ a situation that might suggests that *E. faecalis* can spread between animals and humans through the food chain. Finally, it is important to highlight that PCR-based epidemiological analyses must be taken into consideration that allelic forms of a given determinants can be present in the population, which are not detected by using the standard conditions (as in the case of Type II allele of *bcrABDR*. These situations may underscore the estimations on the prevalence of some resistance genes in bacterial populations).

## Materials and Methods

### Bacterial strains and susceptibility testing

A total of 109 *E. faecalis* were isolated from 181 faecal swabs of healthy chickens. The samples were collected from 11 farms in six provinces of China (Guangdong, Hainan, Guangxi, Hunan, Jiangxi and Zhejiang) in May 2014 (Table S1). The susceptibilities of *bcrABDR*-positive isolates to bacitracin and to other antimicrobials (tetracycline, gentamycin, kanamycin, erythromycin, streptomycin, and florfenicol) were determined by the agar dilution method following the guidelines of the Clinical and Laboratory Standards Institute (CLSI)^24^,^25^. *E. faecalis* ATCC 29212 was used as a quality control strain. Bacterial DNA was extracted using a DNA extraction kit (Omega, USA) following the manufacturer’s instructions. The presence of *bcrABDR* was determined by PCR and further sequencing of the amplicons. Primer walking was performed to analyze more in detail the different *bcrABDR* and surrounding structures in the selected strains. The primers used in this study were presented in Table S2.

### Molecular typing

Pulsed-field gel electrophoresis (PFGE) was performed using the CHEF-MAPPER System (Bio-Rad Laboratories) as described previously^26^ to analyze the clonality of the *E. faecalis* strains carrying *bcrABDR*. PFGE clusters were analyzed with Bio Numerics (Applied Maths, Sint-Martens-Latem, Belgium). Dendrograms were generated using the Dice similarity coefficient, and the analogical values to categorize identical PFGE types cut-off was fixed at 90%. Further determination of clonality was performed by Multi Locus Sequence Typing (MLST), following the protocol described at http://efaecalis.mlst.net.

### Conjugation and transformation analysis

Conjugation and transformation experiments of the *bcrABDR* positive strains were performed as described previously, using *E. faecalis* JH2-2 (Rif^r^) as the recipient strain^27^,^28^. Putative transconjugants and transformants were selected on brain heart infusion agar containing 25 μg/ml fusidic acid, 50 μg/ml rifampicin and 100 μg/ml bacitracin. *bcrABDR* positive transconjugants and transformants were detected by PCR, using the primers described in Table S2.

### Plasmid characterization

To estimate the size of the plasmids harboring *bcrABDR* in the *bcrABDR-*positive strains (37 isolates and 7 transconjugants), whole-cell DNA was separated by PFGE after treatment with S1 nuclease (TaKaRa). Afterwards, the plasmids carrying *bcrABDR* were identified by Southern blot using a *bcrB* probe.

A *bcrB*-positive plasmid from transconjugant JEF123 was extracted using the Qiagen plasmid maxi kit (Qiagen, Valencia, CA). Sequencing was performed using the Miseq platform (Illumina, San Diego, CA) with a 500-bp paired-end library, and assembled by SOAPdenovo^29^. The gaps between the contigs were closed by PCR and respective amplicons were sequenced. Gene prediction and annotation were performed using the RAST tools^30^. The sequence comparison and map generation was performed using BLAST (http://blast.ncbi.nlm.nih.gov) and BRIG^31^.

### Nucleotide sequence accession number

The nucleotide sequence of the plasmid pEF123 containing *bcrABDR*, has been deposited in GenBank under the accession number KX579977.

## Additional Information

**How to cite this article**: Chen, M.-Y. *et al*.Multilevel selection of *bcrABDR*-mediated bacitracin resistance in *Enterococcus faecalis* from chicken farms. *Sci. Rep.* **6**, 34895; doi: 10.1038/srep34895 (2016).

## Supplementary Material

Supplementary Information
