# Supplementary material for: Multilevel selection of bcrABDR-mediated bacitracin resistance in Enterococcus faecalis from chicken farms
Source: Sci Rep. 2016 Oct 12;6:34895. doi: 10.1038/srep34895 (PMC5059624; doi:10.1038/srep34895)
Supplement: Supplementary Information [file srep34895-s1.pdf]

Supplementary data

Multilevel selection of *bcrABDR*-mediated bacitracin resistance in *Enterococcus faecalis* from chicken farms

Mu-Ya Chen[1,2], Felipe Lira[3], Hua-Qing Liang[1,2], Rui-Ting Wu[1,2], Jia-Hong Duan [1,2], Xiao-Ping Liao[1,2], José L. Martínez[3], Jian Sun[1,2*] and Ya-Hong Liu[1,2*]

[1.]National Risk Assessment Laboratory for Antimicrobial Resistance of Animal Original Bacteria, South China Agricultural University, Guangzhou, China

[2.]Guangdong Provincial Key Laboratory of Veterinary Pharmaceutics Development and Safety Evaluation, South China Agricultural University, Guangzhou, China

[3.]Centro Nacional de Biotecnología. CSIC. Darwin 3. 28049-Madrid. Spain.

* Corresponding author:

Ya-Hong Liu, Ph.D, College of Veterinary Medicine, National Reference Laboratory of Veterinary Drug Residues, South China Agricultural University, Guangzhou, 510642, China. Email: lyh@scau.edu.cn. Tel: +86 2085287189, Fax: +86 2085284896

Jian Sun, Ph.D, College of Veterinary Medicine, National Reference Laboratory of Veterinary Drug Residues, South China Agricultural University, Guangzhou, 510642, China. Email: jiansun@scau.edu.cn.

Address correspondence to Ya-Hong Liu, lyh@scau.edu.cn, or Jian Sun, jiansun@scau.edu.cn.

Table S1. Information on the *E. faecalis* isolates used in this study

Souse of isolates	Guangdong	Hainan	Guangxi	Hunan	Zhejiang	Jiangxi	Total
No. of farms	5	1	2	1	1	1	11
No. of faecal swabs	89	16	38	11	6	21	181
No. of isolates	48	11	26	6	4	14	109
No. of *bcrABDR*-positiveisolates(%)	11(22.9%)	4(36.3%)	15(57.6%)	5(83.3%)	2(50%)	0(0%)	37(33.9%)

Table S2. Oligonucleotide primer pairs used in this study

**Gene**	**Primer designation**	**Sequence (5'-3')**	**Product size(bp)**	**Reference or source**
bcrA	P1	5’ -CCGCAATGAAAATGATGTTG-3’	584	1
	P2	5’ -TGCGGCTATCTTACCATCTG-3’		
bcrB	P3	5’ -AAAGAAACCGACTGCTGATA-3’	489	1
	P4	5’ -GCTTACTTGTATAGCAGAGA-3’		
bcrD	P5	5’ -GCGAAGCGTTTAAGGAAATG-3’	482	1
	P6	5’-TGGCACAGCAAGAAAGAATG-3’		
bcrR	P7	5’- TAACGCAGGAACAACTTGC-3’	461	1
	P8	5’ - CAAAGCGGTAATGGTGAGG-3’		
bcrA-1	P9	5' AAATCTTACGAAGCGATACGG 3'	812	This study.
	P10	5' CATCTATCCTTGTCACGAGCA 3'		
bcrD-1	P11	5' ACACTTAATATGAGCGAAGCA 3'	552	This study.
	P12	5' CACTTGTAAAATCAAACCCGAA 3'		
Inverse	P13	5' TCGCAGTACCTACTATGCTTG 3'	1909	This study.
PCR	P14	5' CGGAAGAAATATAAACGCAAC 3'		

Table S3. The antimicrobial susceptibilities of the wild-type strains, transconjugants, and recipient strains in this study.

**Strains**	**MICs (mg/L)**					
	BAC	ERY	FFC	TET	STR	KAN
**127**	>256	>256	4	128	>256	>256
**J127**	>256	>256	4	128	>256	>256
**7**	>256	>256	64	256	>256	>256
**J7**	>256	>256	128	256	>256	>256
**174**	>256	>256	128	128	>256	>256
**J174**	>256	>256	4	<2	>256	>256
**8**	>256	>256	64	128	>256	>256
**J8**	>256	>256	4	128	>256	>256
**129**	>256	>256	4	64	>256	>256
**J129**	>256	>256	4	128	>256	>256
**105**	>256	>256	64	128	>256	>256
**J105**	>256	>256	4	<2	>256	>256
**123**	>256	>256	128	128	>256	>256
**J123**	>256	>256	128	128	>256	>256
**JH2-2**	32	<2	4	<2	32	32

BAC, bacitracin; STR, streptomycin; ERY, erythromycin; TET, tetracycline; FFC, florfenicol; KAN, kanamycin

Strains of 127, 7, 174, 8, 129, 105, 123 and its transconjugants J127, J7, J174, J8, J129, J105, J123, respectively.

Figure S1. Location of the locus *bcrABDR* in the 37 *E. faecalis* strains, and corresponding transconjugants.

(a)

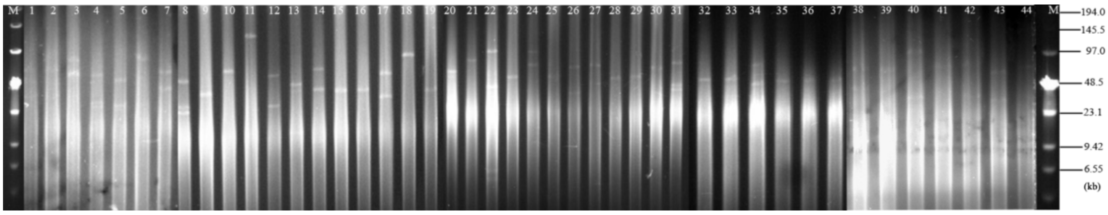

(b)

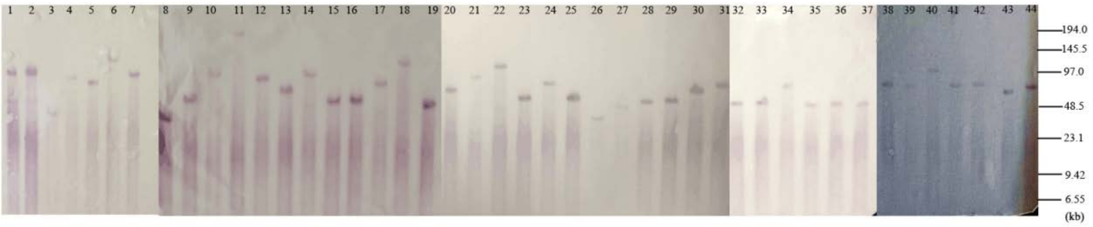

Figure S1. Location of the locus *bcrABDR* in the 37 *E. faecalis* strains, and corresponding transconjugants.
(a) S1-PFGE of the *bcrABDR*-positive strains, (b) subsequent southern hybridization with *bcrB*-specific probe. Lanes: M, Low Range PFG Marker; 1, EF178; 2, EF179; 3, EF176; 4, EF174; 5, EF129; 6, EF25; 7, EF127;8, EF15; 9, EF52; 10, EF54; 11, EF171; 12, EF8; 13,EF169; 14, EF168; 15 EF53; 16,EF59; 17,EF77; 18, EF109; 19,EF136; 20, EF51; 21, EF14; 22, EF7; 23, EF118; 24, EF123; 25, EF62; 26, EF21; 27, EF24; 28, EF39; 29, EF61; 30, EF96; 31, EF105; 32, EF57; 33, EF64; 34, EF80; 35, EF137; 36, EF138; 37, EF134; 38, JEF127; 39, JEF174; 40, JEF7; 41, JEF105; 42, JEF8; 43, JEF129; and 44, JEF123.

Figure S2. Genetic structure of the plasmid pEF123.

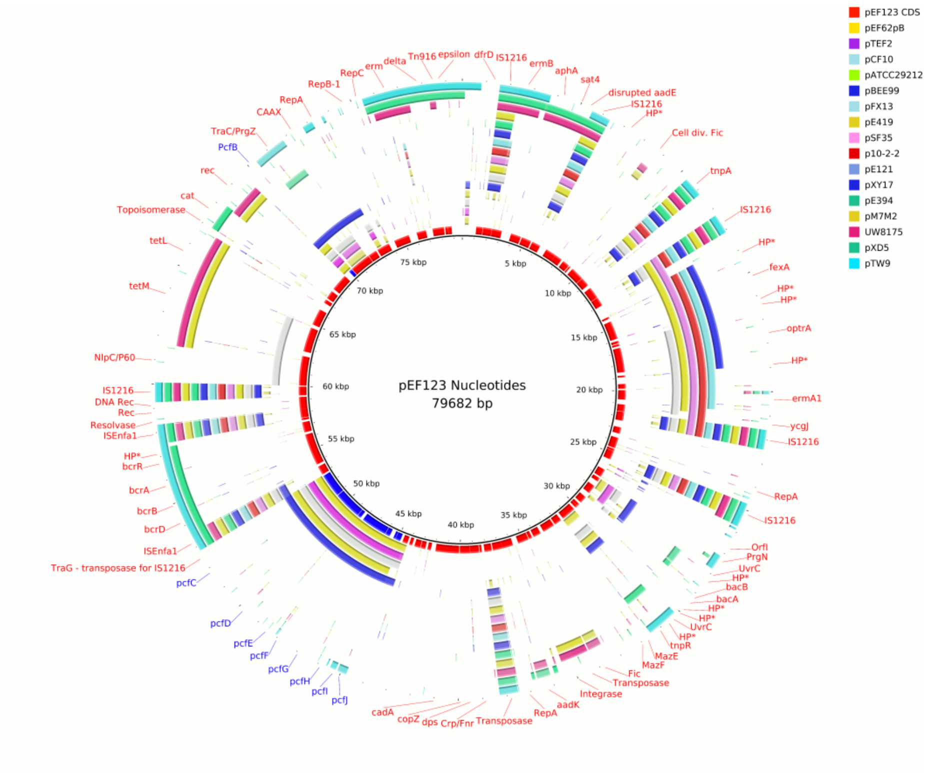

Figure S2. Genetic structure of the plasmid pEF123. The structure of the plasmid pEF123, sequenced in the current work is presented in comparison with other *E. faecalis* plasmids. As shown, share different features with other enterococcal plasmids. To note here that several of the analyzed plasmids harbour IS*Enfa1*, highlighting the relevant role that these insertion sequences may have in the evolution of plasmids in Enterococci.

Reference

1. Matos, R., Pinto, V. V., Ruivo, M. & Lopes, M. D. S. Study on the dissemination of the *bcrABDR* cluster in *Enterococcus* spp. reveals that the BcrAB transporter is sufficient to confer high-level bacitracin resistance. *Int J Antimicrob Agents.* **34**, 142-147, doi:DOI 10.1016/j.ijantimicag.2009.02.008 (2009).
